# AIMD-Chig: Exploring the conformational space of a 166-atom protein *Chignolin* with *ab initio* molecular dynamics

**DOI:** 10.1038/s41597-023-02465-9

**Published:** 2023-08-22

**Authors:** Tong Wang, Xinheng He, Mingyu Li, Bin Shao, Tie-Yan Liu

**Affiliations:** 1 Microsoft Research AI4Science, Beijing, China; 2 Work done during an internship at Microsoft Research AI4Science, Beijing, China; 3 State Key Laboratory of Drug Research and CAS Key Laboratory of Receptor Research and, Shanghai Institute of Materia Medica, Chinese Academy of Sciences, Shanghai, China; 4 University of Chinese Academy of Sciences, Beijing, China; 5 Department of Pathophysiology, Key Laboratory of Cell Differentiation and Apoptosis of Chinese Ministry of Education, Shanghai Jiao Tong University, School of Medicine, Shanghai, China

**Keywords:** Computational biology and bioinformatics, Chemistry

## Abstract

Molecular dynamics (MD) simulations have revolutionized the modeling of biomolecular conformations and provided unprecedented insight into molecular interactions. Due to the prohibitive computational overheads of *ab initio* simulation for large biomolecules, dynamic modeling for proteins is generally constrained on force field with molecular mechanics, which suffers from low accuracy as well as ignores the electronic effects. Here, we report AIMD-Chig, an MD dataset including 2 million conformations of 166-atom protein *Chignolin* sampled at the density functional theory (DFT) level with 7,763,146 CPU hours. 10,000 conformations were initialized covering the whole conformational space of *Chignolin*, including folded, unfolded, and metastable states. *Ab initio* simulations were driven by M06-2X/6-31 G* with a Berendsen thermostat at 340 K. We reported coordinates, energies, and forces for each conformation. AIMD-Chig brings the DFT level conformational space exploration from small organic molecules to real-world proteins. It can serve as the benchmark for developing machine learning potentials for proteins and facilitate the exploration of protein dynamics with *ab initio* accuracy.

## Background & Summary

Molecular dynamics (MD) simulations capture the behaviors of biomolecules in full atomic detail, serving as a computational microscope for molecular biology^1,2^. With MD, the conformation ensemble of biomolecules can be observed, which leads to a deeper understanding of the biomechanism and targeting drug design^3–5^. Based on molecular mechanics, MD simulations employ force fields to describe biomolecular dynamics as atoms with fixed connections and properties^6^. Therefore, the internal interactions are treated with harmonic or periodic functions, while the parameters to describe non-bonded interactions were fitted to pairwise additive Coulomb and van der Waals potentials. Such parameters are derived from estimations and are commonly assumed to be constant, even for different proteins or conformations, which do not accurately reflect the laws and phenomena of the real world^7,8^. For example, the electrostatic interactions are described by fixed point charges located at the atom centers while polarization effects and the electrostatic effects between bonded atoms are neglected^9,10^. Consequently, the challenges of modeling the motions of atoms have historically limited the accuracy and reliability of MD simulations^1,11^.

Quantum mechanics (QM) has been widely applied to accurately describe the properties and behaviors of molecules by considering the motions of electrons. With Born-Oppenheimer (BO) approximation^12^, the wave functions of atomic nucleus and electron can be treated respectively, thereby decreasing the complexity of wave functions and permitting explicit *ab initio* calculations from electron effects^13^. BO approximation describes the system energy as the function of nuclear Cartesian coordinates^12^. Furthermore, Hartree-Fock (HF) and density functional theory (DFT) were proposed to simplify the calculations for electron motions and have been widely used for small chemical molecules^14–16^. However, due to the time complexity is *O*(*N*^3^) to *O*(*N*^4^), it is computationally prohibitive to simulate biomolecules with the laws of quantum mechanics^17^.

To balance the accuracy and efficiency of molecular dynamics simulation, machine learning potentials have become increasingly attractive with the development of deep learning^18^. Essentially, a force field is derived from fitting a potential function that describes the energy of the whole system and the force on each atom upon specific Cartesian coordinates^2,6,19^. With deep learning, arbitrarily complex energy functions can be learned in a data-driven way. Thus, the accuracy of machine learning potential could reach chemical accuracy upon enough and accurate data^20^. Furthermore, highly parallel calculations on GPUs save a lot of time consumption for machine learning potential, leading it feasible for large molecules^21^. Therefore, several datasets are built at the DFT level for machine learning potential design, e.g., MD17^13^, revised MD17^22^, QM7^23^, QM9^24^, ISO17^25^, and so on. However, such datasets are designed for small organic molecules. Recently, MD22 was proposed to provide energies and forces for biomolecules with tens to hundreds of atoms^26^. Whereas there are only tens of thousands of samples for each kind of molecule that starts from a single structure, the MD simulation and the generated dataset are far from full exploration of the whole conformational space, which may lead to the machine learning potential under-fitting the potential energy surface that cannot be well modeled directly.

Significant progress has been made in this field, particularly with the advent of models such as ANI^27^ and TensorMol^28^. These models strive to broaden the sampling of chemical environments by generating specific descriptors for each atom’s environment. Coupled with active learning, advanced ANI potentials are capable of sampling lengthy MD simulation trajectories on small molecules and proteins, as demonstrated in the COMP6 datasets^29^. However, these models employ classical MM to represent long-range interactions, which could potentially lead to inaccurate descriptions during protein folding and functioning simulations^30,31^. *Ab initio* simulations of large molecules with varying conformations can furnish the requisite data for training a “residual” model for energy and force prediction, enabling it to model long-range interactions with *ab initio* accuracy. It is imperative, therefore, to address these existing limitations to further augment the efficiency and applicability of machine learning potentials in bio-simulations.

**Fig. 1:**
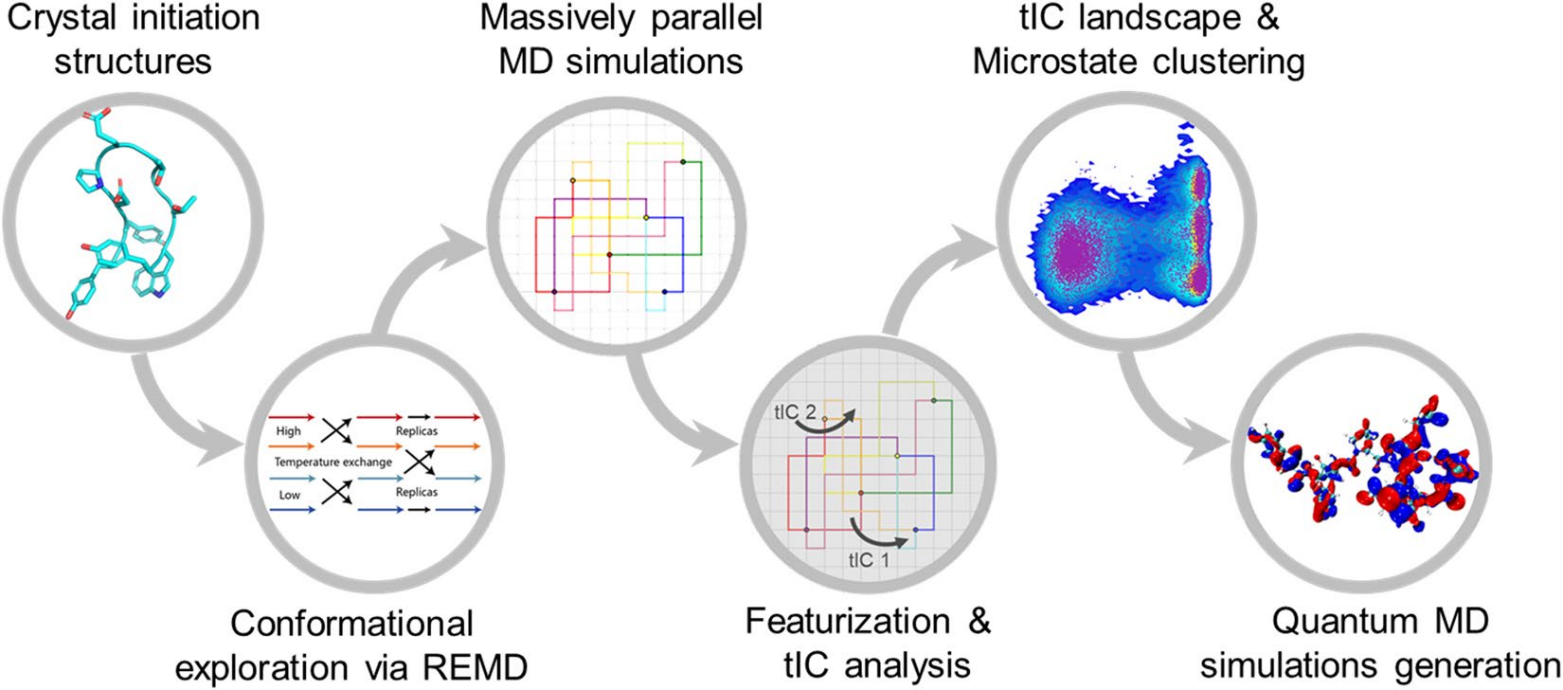
The overall pipeline to build the AIMD-Chig dataset. The simulations started from the crystal structure of *Chignolin* (PDB ID: 5AWL). We first explored the conformation sampling via REMD on 8 different temperatures. Then, conventional MD simulations from 100 representative structures derived from REMD were conducted. After such simulations, we projected the raw coordinate space into a 6-D space according to the tIC analysis. On the projected space, we extracted 10,000 cluster centers as the initial structures for *ab initio* MD simulations and ran 225 steps of *ab initio* MD with M062X/6-31 G* setting for each. The last 200 steps of each *ab initio* MD simulation were reported in the dataset.

In this work, we propose AIMD-Chig, a benchmark dataset to fully explore the conformational space of a 166-atom protein *Chignolin* at the DFT level. The dataset consists of 2 million samples with different conformations of *Chignolin*, and the corresponding potential energies and forces are calculated with M06-2X/6-31 G*. The pipeline to construct AIMD-Chig is illustrated in Fig. 1. We first ran 10 ns replica exchange molecular dynamics (REMD) simulations and 100 μs conventional MD simulations to sample the full conformational space of *Chignolin*. Then, we applied time-lagged component (tIC) analysis to construct the free energy landscape and capture different conformations. 10,000 conformations on the energy landscape were picked as the initial structures (termed “anchors”) for *ab initio* MD simulations at DFT level. For each anchor, we ran 225 fs *ab initio* MD simulations with a time step of 1 fs and extracted all conformations after 25 fs to build the dataset. AIMD-Chig not only serves as the benchmark for developing machine learning potentials but also sheds a light on the exploration of protein dynamics with *ab initio* accuracy.

## Methods

The overall pipeline of the dataset construction method is shown in Fig. 1. To cover the conformational space of *Chignolin* completely and accurately, we first adopted REMD and conventional MD simulations to sample the conformations. Then, from 10,000 anchors on the free energy landscape for *Chignolin* conformations, we ran 225 steps of *ab initio* MD simulations. Such a sampling process leveraged both molecular mechanics to cover different conformations and *ab initio* simulations to provide an accurate estimation of energy, force, and coordinates.

### MD simulations

The initial structure of *Chignolin* for MD simulations was obtained from Protein Data Bank (PDB ID: 5AWL)^32^. The protein (with the sequence “YYDPETGTWY”) was then solvated in a generalized Born implicit solvent model^33^. The FF19SB force field was applied to describe the interactions between atoms^10^. After energy minimization, the system encountered 200 ps equilibration and 10 ns replica exchange molecular dynamics (REMD)^34^ production runs under 8 different temperatures, i.e., 300 K, 400 K, 500 K, 600 K, 700 K, 800 K, 900 K, and 1000 K. The exchange of temperatures happened per 2 ps.

After REMD, we projected the trajectories to a two-dimensional surface according to two inter-atomic distances. One was the distance between atom O on residue Y2 and atom N on residue G7. The other one was the distance between atom O on residue E5 and atom N on residue T8. We then clustered all conformations on the free energy landscape and picked 100 structures as the representative conformations during REMD. These structures were used as the input for conventional MD simulations. They were solvated in a TIP3P water^35^ box with a buffer of 10 Å. Then, 2 Na+ ions were added to the systems for neutralization and 0.15 mol∙L^−1^ NaCl was also added to the solvents.

The systems were first minimized by 45,000 cycles. Next, the systems were heated to 300 K in 300 ps and equilibrated for 700 ps. Finally, each system encountered 1 μs NPT production MD run, accumulating 100 μs simulation time from different initial structures. Long-range electrostatic interactions were treated by the Particle mesh Ewald algorithm^36^. A cutoff of 10 Å was employed for short-range electrostatic and van der Waals interactions. The SHAKE algorithm was applied to restrain the bond with hydrogens^37^. MD simulations were performed by Amber20 software^38^.

### Anchor selection

From the trajectories of MD simulations for *Chignolin*, the time-lagged independent component (tIC) analysis was applied to decrease the dimensions of the conformational space. The tIC analysis was specially designed for capturing slow dynamics during simulations^39^, and thus it has been widely used to extract representative structures from a large number of simulation trajectories.

The coordinates of *Chignolin*’s conformations during simulations were first aligned to the crystal structure. Then, the aligned raw coordinates were employed for tIC analysis and dimensional reduction. The lag time for tIC analysis was set to 20 ns and the conformational space was decreased to 6 dimensions. Then, the minibatch k-means algorithm was used to extract 10,000 representative structures on the tIC surface. These structures were defined as anchors for the following *ab initio* MD simulations.

### *Ab initio* MD simulations

All 10,000 anchors of *Chignolin* were run *ab initio* MD simulations at DFT level using ORCA 4.2.1 packge^40^. M06-2X functional in conjunction with 6–31 G* basis set was employed for the calculation^41^. The combination M06-2X/6–31 G* presents a good balance between the accuracy and the computational cost, takes the weak interactions among atoms into consideration, and has been widely used for biomolecules^42–44^. We adopted normal self-consistent-field (SCF) convergence (1 × 10^−6^ a.u. energy difference between two successive iterations) and set the maximum iterations to 300. For each anchor, the step of simulations was set to 1 fs, and 225 simulation steps were run. A temperature of 340 K during the simulation was controlled via Berendsen thermostat with a τ value of 10 fs. The production runs were made on 2,000 computational nodes where each computational node has 36 Intel Xeon Platinum 8272CL CPU cores. Since *Chignolin* has 166 atoms, the computational time for each simulation step is about 6 minutes on a node. In total, the *ab initio* MD simulations took 7,763,146 CPU hours. After *ab initio* simulations, the first 25 frames of each trajectory were discarded due to fluctuated temperature, and the coordinates, potential energy, and atomic forces of the remaining frames were extracted and reported in our dataset.

### Analysis and validation

We collected the calculation time, kinetics, and potential energy for each simulation step and evaluated their variations during the last 200 fs simulations. For all points, the distributions of potential energy, the norm of force, and the forces in the x, y, and z respective directions were also depicted.

To confirm the sampling reasonability of MD simulations and anchor selection, we clustered all conformations from MD simulations with 200, 500, 1000, 2000, 5000, and 10000 clusters by the minibatch k-means algorithm respectively and plotted the distributions of different numbers of anchors on the tIC surface. As a comparison, we also plotted the 2 million snapshots from *ab initio* MD simulations on the same potential energy surface. On the tIC surface, the relative energy values were calculated by the potential of mean force (PMF). The PMF energy was given by Eq. (1):

1 $${\Delta }G(x,y)={k}_{B}Tlng(x,y)$$

where k_B_ means the Boltzmann constant, *T* is the temperature of the system and *g*(x, y) represents the normalized joint probability distribution. The minimum energy value was set to zero. 150 bins were applied to generate the landscape in both the x and y directions.

For the validation of our algorithm, DLPNO-CCSD(T) function^45^ with cc-pVTZ/C auxiliary basis^46^ was applied on 200 snapshots from the simulations for single point energy evaluation. Referring to the structure with the smallest index, we calculated the relative potential energy values for each structure and compared them with M06-2X/6-31 G* results. Furthermore, we also did geometry optimization for both unfolded and folded structures by revPBE-D3(BJ)/def2-TZVP^47–49^ and M06-2X/6–31 G*, respectively. Then compared the endpoint structure.

From our dataset, we trained VisNet model with subsets of our dataset, utilizing 1%, 5%, 30%, and the entirety of the data. For the purpose of running simulations, we primarily employed the model trained on the full dataset. The partitioning of the dataset was carried out using a scaffold split method. The training parameters were maintained consistent with those detailed in the original publication^21^. Systems were firstly minimized for 15,000 cycles, then generally heated to 300 K in NVT environment in 300 ps. At last, the systems were equilibrated for 700 ps in NPT environment whose pressure was 1 atm. We executed 10 independent simulation runs within the Atomic Simulation Environment (ASE). Using Amber QM-MM, a TIP3P water box was used to encapsulate the entire structure and the interactions for water-water and water-protein were evaluated by MM. The 20,000 steps of simulations were finished under 300 K NVT condition with a timestep of 0.5 fs.

To evaluate the accuracy of different approaches for MD simulation, from 200 snapshots from the simulations, we calculated their potential energies and atomic forces by molecular mechanics (Amber FF19SB)^10^, semi-empirical approach comprising the NDDO (Neglect of Diatomic Differential Overlap) approximation-based (Parametric Method 3, PM3)^50^, DFT approximation-based methods (DFTB)^51^, as well as HF with 6–31 G* basis. Then, referring to the structure with the lowest energy, we calculated the relative potential energy values for each structure. The mean force error represents the average of the difference of the forces on each atom. The max force error represents the max difference of the forces on all atoms, then the value was averaged on 200 snapshots. The relative potential energies and atomic forces calculated by different approaches were compared with our *ab initio* simulation approach.

## Data Records

### Data structure

**Fig. 2:**
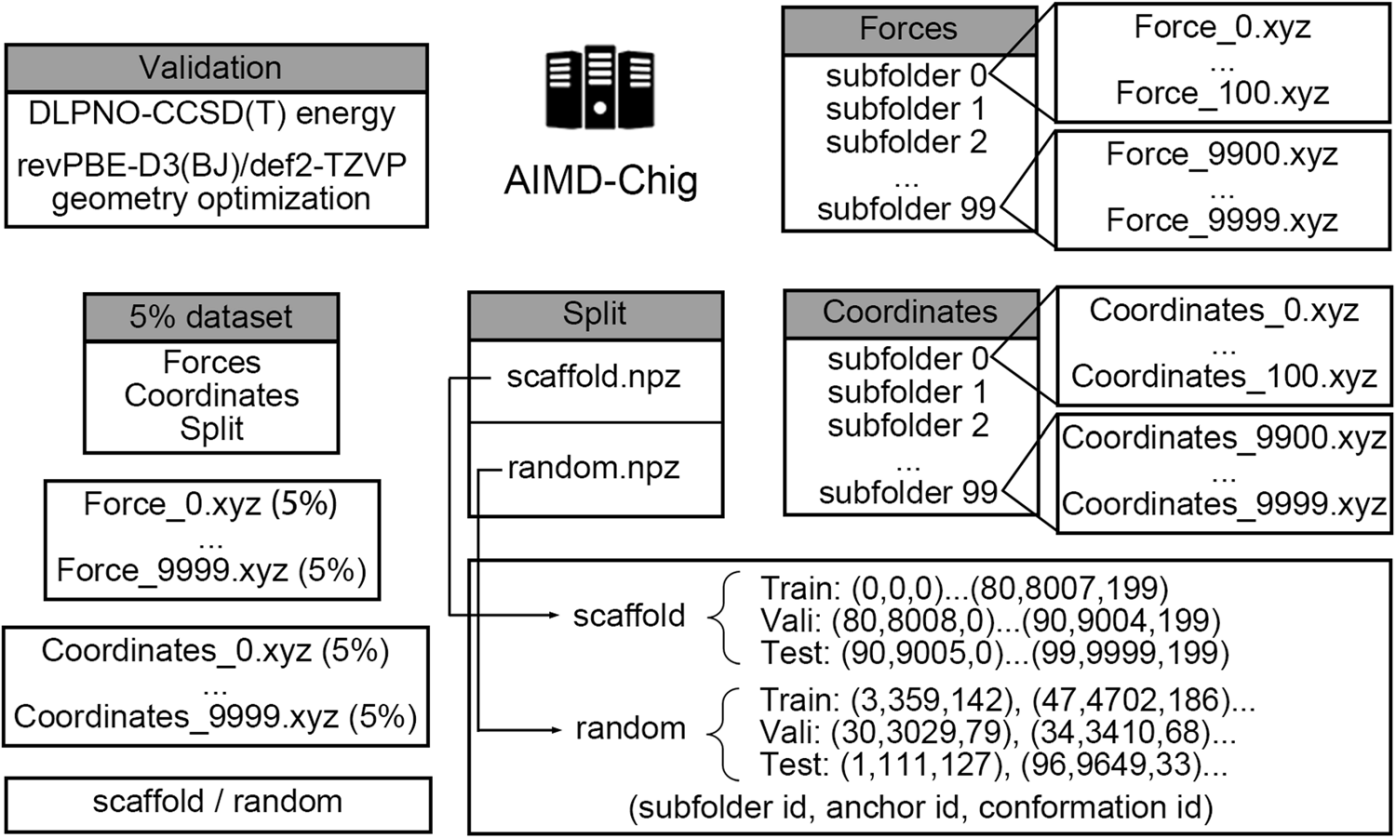
Schematic description of the structure of the AIMD-Chig dataset. The directories of “Forces” and “Coordinates” have 100 subfolders including force and coordinate information as “xyz” format files, respectively. In each “xyz” format file, potential energy values were shown in the second line. The directory of “Split” presents the training, validation and test sets split scheme. Two kinds of split modes, scaffold split and random split were both provided with respective “npz” files. In each “npz” file, the index is shown as lists. In each list, item 0 is the index of the corresponding subfolder of “Forces” and “Coordinates” directories, item 1 is the index of the anchor and item 2 is the index of the conformation that was simulated starting from the anchor. We also added the validation of M06-2X algorithm and a smaller 5% dataset for easy test in our dataset.

The AIMD-Chig dataset has been deposited in figshare under accession number 10.6084/m9.figshare.22786730^52^. The data were separately packed into several directories in the compressed zip files. ‘Forces’ consists of the atomic forces for each conformation during simulations while ‘Coordinates’ folder consists of the corresponding coordinates with the “xyz” format. Potential energy values were shown in both force and coordinate xyz files. All these data have a precision of 10 digits. The units for force, coordinate, and energy were Hartree/Å, Å, and Hartree, respectively. For the structures that did not reach SCF convergence during simulations, the xyz files for coordinates and forces were not shown. Thus, in each folder, there are 9,955 files corresponding to the 10,000 anchors with indices ranging from 0 to 9999. The files were archived into 100 subfolders where each subfolder contained 100 anchors in turn. To facilitate ML potential training and evaluation, we also provided two kinds of data split, namely “scaffold” and “random”. In the scaffold split mode, we divided training, validation, and test indexes according to anchors. In other words, samples in the training, validation and test datasets were simulated from different initial structures. In contrast, the random split mode mixed the data altogether and randomly divided the 3 datasets. We also provide a smaller dataset with 5% (10 snapshots for each AIMD simulation run) data for a quick evaluation as well as materials for validation on calculation approaches. Figure 2 shows a schematic representation of the AIMD-Chig data structure that describes the path of all files provided.

### Data statistics

**Fig. 3:**
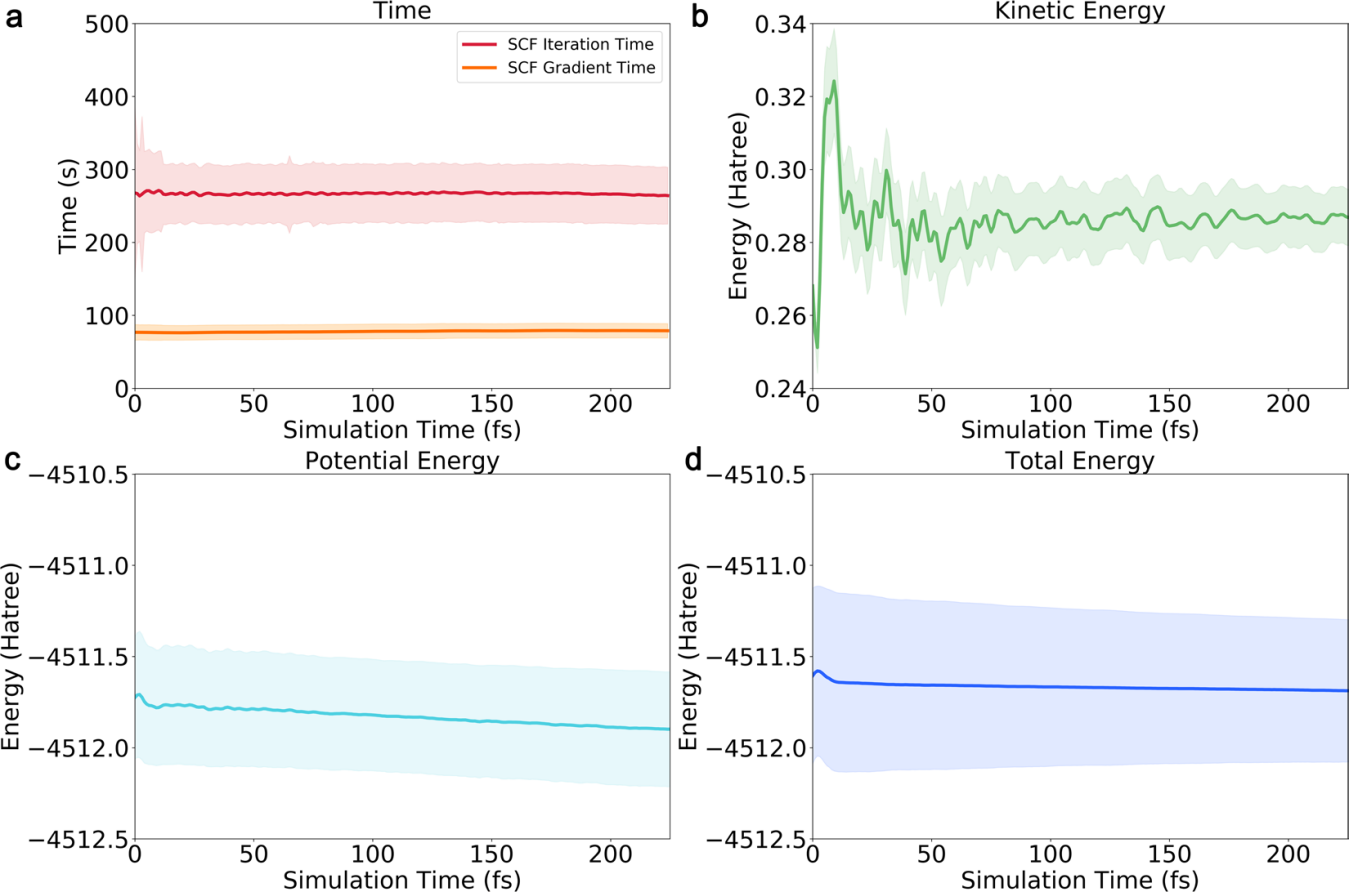
The time-course curves of different properties of *ab initio* molecular dynamics simulations for *Chignolin*. (**a**) The calculation time of SCF iteration and gradient evaluation; (**b**) The kinetic energies; (**c**) The potential energies; (**d**) The total energies. From (**a**–**d**), the average values are shown in line while the ranges for the same simulation step of all anchors are shown in shadow.

The time-course curves of different properties during *ab initio* molecular dynamics simulations are shown in Fig. 3. We first analyzed the most time-consuming procedures, i.e., SCF iterations and gradient calculation. As shown in Fig. 3a, the average time consumptions of SCF iterations and gradient calculations were 267.78 ± 40.24 seconds and 78.15 ± 10.07 seconds, respectively, which indicates that the time consumptions for each simulation step are fluctuant. The kinetic energy has little fluctuations in all simulation steps, showing the temperature in the *ab initio* simulation kept constant (Fig. 3b). The average value of it was 0.286 ± 0.010 Hartree. The potential energy accounts for most of the total energy (Fig. 3c,d). Both the potential energy and the total energy have a declining trend during the simulations. The decrease of energy values during simulations meets the criteria that MD simulation tends to lead structures to stable ones in energy basins. The average values of potential energy and total energy were −4511.54 ± 0.079 Hartree and −4511.25 ± 0.080 Hartree, respectively.

**Fig. 4:**
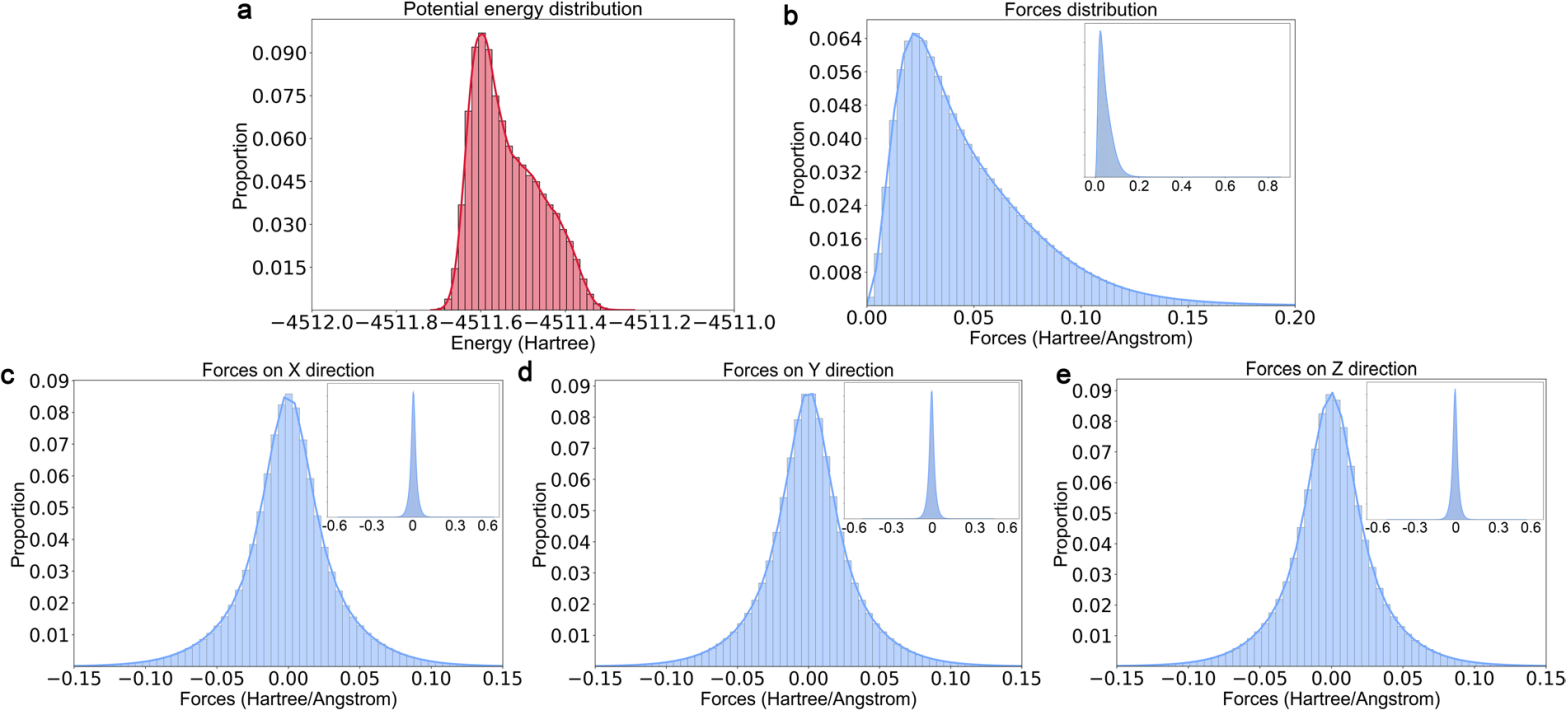
The distributions of the potential energy and atomic forces of all samples in AIMD-Chig. (**a**) the potential energy; (**b**) the modulus of atomic forces; (**c**–**e**) the atomic forces in each dimension. For better visualization, the dominant data distributions in panels (**b**–**e**) are shown while the whole data distributions are shown as a subplot in the upper right region for each panel.

The statistics for all samples in AIMD-Chig are shown in Fig. 4 and Table 1. The potential energy distribution of samples has a peak on the left of the distribution curve with the value of −4511.6 Hartree (Fig. 4a), which is consistent with the decreasing tendency of energies during simulations. The upper and lower bounds for the potential energy have a difference of 0.48 Hartree, reflecting that the energy differences of different conformations can reach hundreds of kcal/mol. As for the atomic forces, the average modulus of forces was 4.67 × 10^−2^ ± 3.20 × 10^−2^ Hartree/Å, which corresponds to the peak in the distribution curve (Fig. 4b). Although the average force is relatively small, the largest force reached 0.857 Hartree/Å, indicating that the conformational changes of proteins permit the existence of a large force. For the distributions of atomic forces in every direction (Fig. 4c–e), all exhibit a gaussian distribution in which the average value was around 0.

**Table 1:** Statistics of the potential energy and atomic forces of the samples in AIMD-Chig.

Properties	Ave.	Std.	Min. value	Max. value
Potential energy (Hartree)	−4511.54	7.88 × 10^−2^	−4511.72	−4511.24
Force modulus (Hartree/Å)	4.67 × 10^−2^	3.20 × 10^−2^	1.73 × 10^−5^	0.857
Force X (Hartree/Å)	2.39 × 10^−4^	3.36 × 10^−2^	−0.702	0.747
Force Y (Hartree/Å)	−2.45 × 10^−5^	3.24 × 10^−2^	−0.630	0.569
Force Z (Hartree/Å)	2.54 × 10^−4^	3.20 × 10^−2^	−0.614	0.646

## Technical Validation

### Validation of conformational sampling diversity

**Fig. 5:**
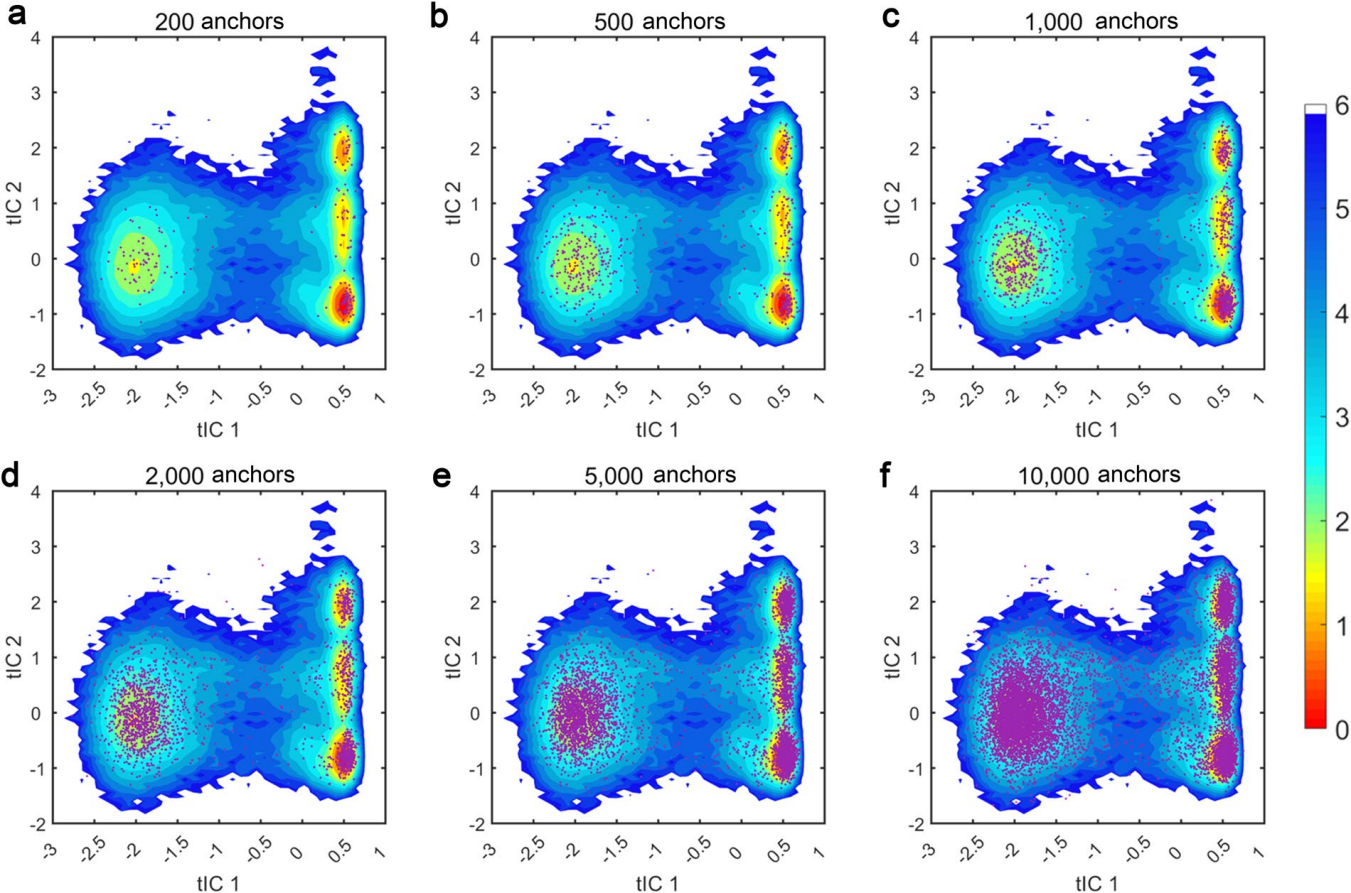
Evaluation of the choices of different numbers of anchors on the free energy landscape of *Chignolin*. From (**a**–**f**), the numbers of anchors increase from 200 to 10,000. Purple points indicate the position of the anchor structure on the free energy landscape. The unit of the relative energy is kcal/mol.

We first evaluated the sampling diversity in the AIMD-Chig dataset. As shown in Fig. 5, we plotted the choices of different numbers of anchors on the free energy surface. On the energy landscape, there exists four energy basins. One is on the left region and the other three are on the right region. The energy basin whose tIC 1 is lower than −1 corresponds to the unfolded structures, while the right three basins (tIC 1 > 0) correspond to the folded states of *Chignolin*. The metastable states are in the middle region (tIC 1 around −0.6). When only 200 anchors from MD simulations were chosen, all conformations were located at the energy basins and no metastable state was sampled (Fig. 5a). When the number of anchors increased to 500, a few conformations in the metastable states began to be sampled (Fig. 5b). It is worth noticing that the sampling in the metastable regions was not promoted when the number of anchors increased from 500 to 5,000 (Fig. 5c–e). Upon 10,000 anchors, the sampling of the metastable domain was significantly enhanced and even some high-energy points on the white background were successfully sampled (Fig. 5f). Therefore, to balance the diversity of conformations and the computational cost, we chose 10,000 anchors as the initial structures for *ab initio* molecular dynamics simulations.

**Fig. 6:**
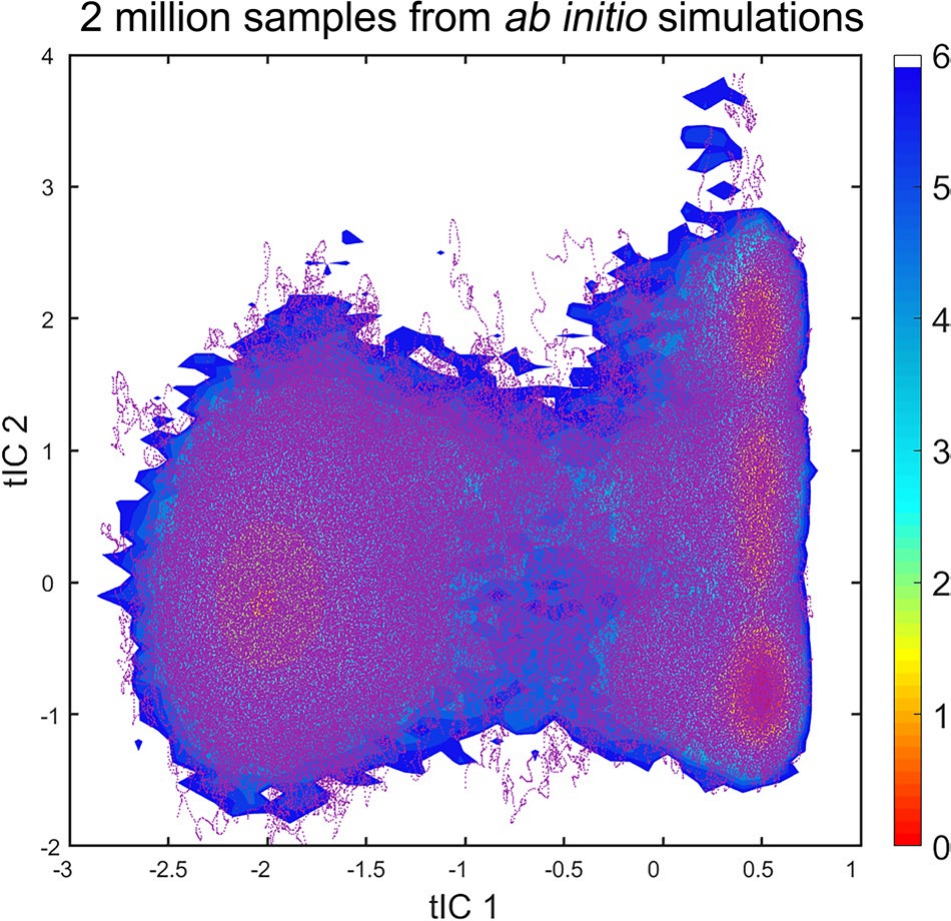
The distribution of 2 million samples from *ab initio* simulations on the free energy landscape of *Chignolin*. Purple points indicate the position of samples. The unit of relative energy is kcal/mol.

As shown in Fig. 6, the 2 million samples were plotted on the free energy landscape of *Chignolin*. Starting from 10,000 anchors, the *ab initio* MD simulations were able to cover different conformations on the potential energy surface. For the energy basins, it is obvious that the purple points covered all the energy basins and most of the metastable states. Therefore, the diversity of conformations in AIMD-Chig was confirmed that it has covered the transitions among different folding and unfolding states of *Chignolin*.

Such a dataset could guide ML potentials in discerning both various long-rang interactions in very different conformations and subtle energy and force differences among similar conformations, enabling localized models to attain *ab initio* level insights into long-range interactions. In addition, any unexplored conformations could be further recruited by active learning during model training, which is complement to the original dataset.

### ML potential trained on AIMD-Chig

To prove the usefulness of AIMD-Chig for ML potential training, we trained a series of ML potentials based on ViSNet, a state-of-the-art equivariant GNN for molecular modeling^21,53^. The dataset was split with the scaffold scheme. We first split two pieces of 10% data from the entire dataset as the validation and test sets, respectively. For the remaining data, we adopted different amounts of data (1%, 5%, 30%, and all) for model training while making evaluations on the same validation and test sets that were independent to all sizes of training sets. The mean absolute error (MAE) on the test set for both energy and force were shown in Table 2.

**Table 2:** Performance of VisNet Model trained on varying data amounts from AIMD-Chig.

Data amount	Energy MAE (kcal/mol)	Force MAE (kcal/mol*Å^−1^)
1%	102.43	3.783
5%	3.782	0.549
30%	1.453	0.280
100%	0.738	0.195

When we used only 1% of the data (2 snapshots per AIMD simulation run), the MAE for energy was 102.43 kcal/mol, and for force, it was 3.783 kcal/mol*Å^−1^. When the data was increased to 5% (10 snapshots per AIMD simulation run), the MAE for energy decreased to 3.782 kcal/mol, and for force, it dropped to 0.549 kcal/mol*Å^−1^. This downward trend continued with 30% of the data (60 snapshots per AIMD simulation run), yielding an energy MAE of 1.453 kcal/mol and force MAE of 0.280 kcal/mol*Å^−1^. Finally, when the entire training dataset was used, the MAE for energy was reduced to 0.738 kcal/mol, and for force, it was 0.195 kcal/mol*Å^−1^. These results indicate that the data from the same simulation trajectories were not redundant but provided significant value in training the ML potentials. In addition, an intelligently selected sub-sampling from the whole dataset could also be made depending on the specific requirements and application contexts.

**Fig. 7:**
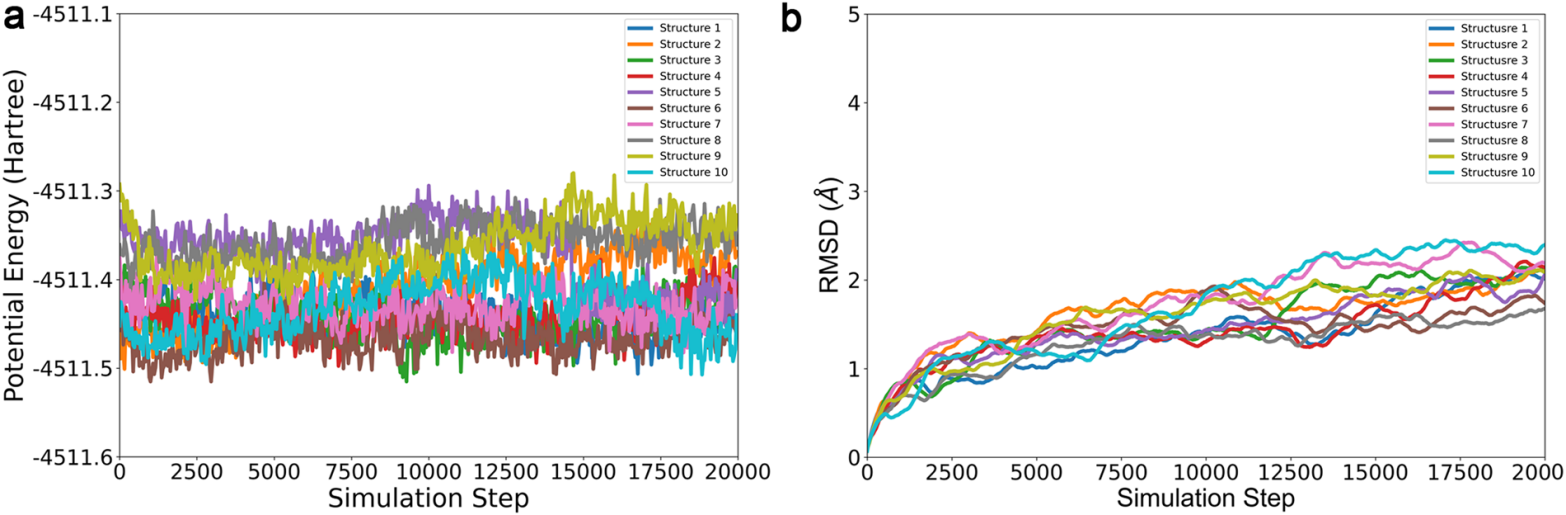
The variation during 10 independent simulations with ML potential. (**a**) the potential energy of *Chignolin*; (**b**) the RMSD of all atoms.

In order to substantiate the utility of our dataset, we implemented such VisNet potential to conduct molecular dynamics simulations from 10 distinct *Chignolin* structures. Each simulation executed 20,000 steps under 300 K NVT conditions, maintaining a timestep of 0.5 fs. The outcome of these simulations, including the fluctuations in protein potential energy and Root Mean Squared Deviation (RMSD) are comprehensively illustrated in Fig. 7. It is evident from the potential energy and RMSD plots (Fig. 7a,b) that all simulations seamlessly completed the designated 20,000 steps and eventually stabilized. In summary, these rigorous evaluations confirm the robustness and reliability of our dataset as a source for training machine learning models, and such models are able to generate stable molecular dynamics simulations.

### Comparison of calculation approaches

**Fig. 8:**
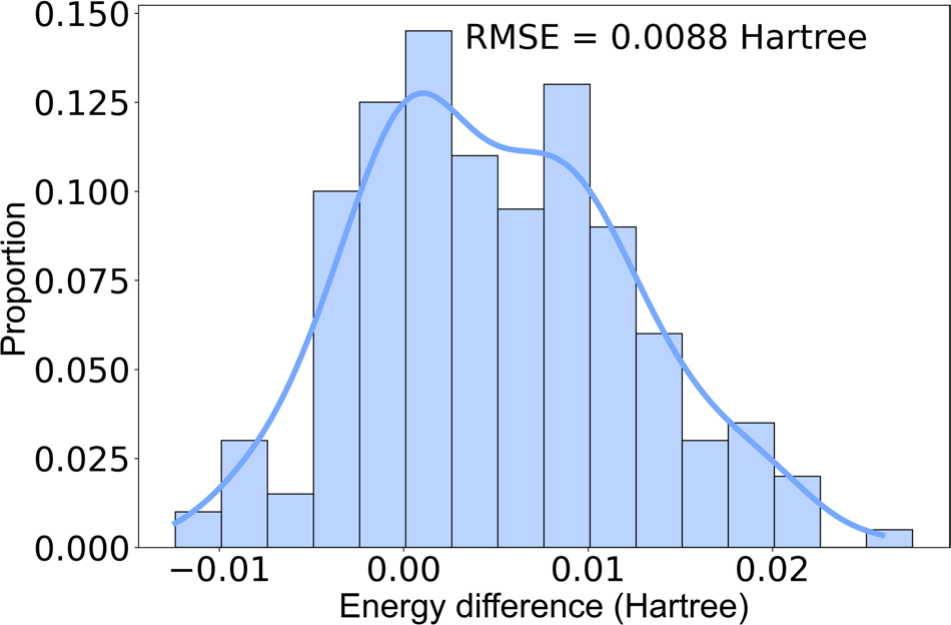
The distribution of DLPNO-CCSD(T) and M06-2X in single point energy evaluation on 200 randomly picked points from AIMD-Chig dataset. The relative energy values were calculated according to the structure with the smallest id in our dataset in the 200 snapshots.

We first compared M06-2X/6-31 G* method with more precise approaches. Primarily, we compared the single point energies of 200 snapshots calculated by DLPNO-CCSD(T) and M062X, respectively. As depicted in Fig. 8, the energies calculated by M06-2X was similar with those calculated by DLPNO-CCSD(T), with a RMSE of 0.0088 Hartree. This suggests that M06-2X characterizes the system with high consistency, akin to the DLPNO-CCSD(T) method, and thus is a reliable method for single point energy calculation with high accuracy.

**Fig. 9:**
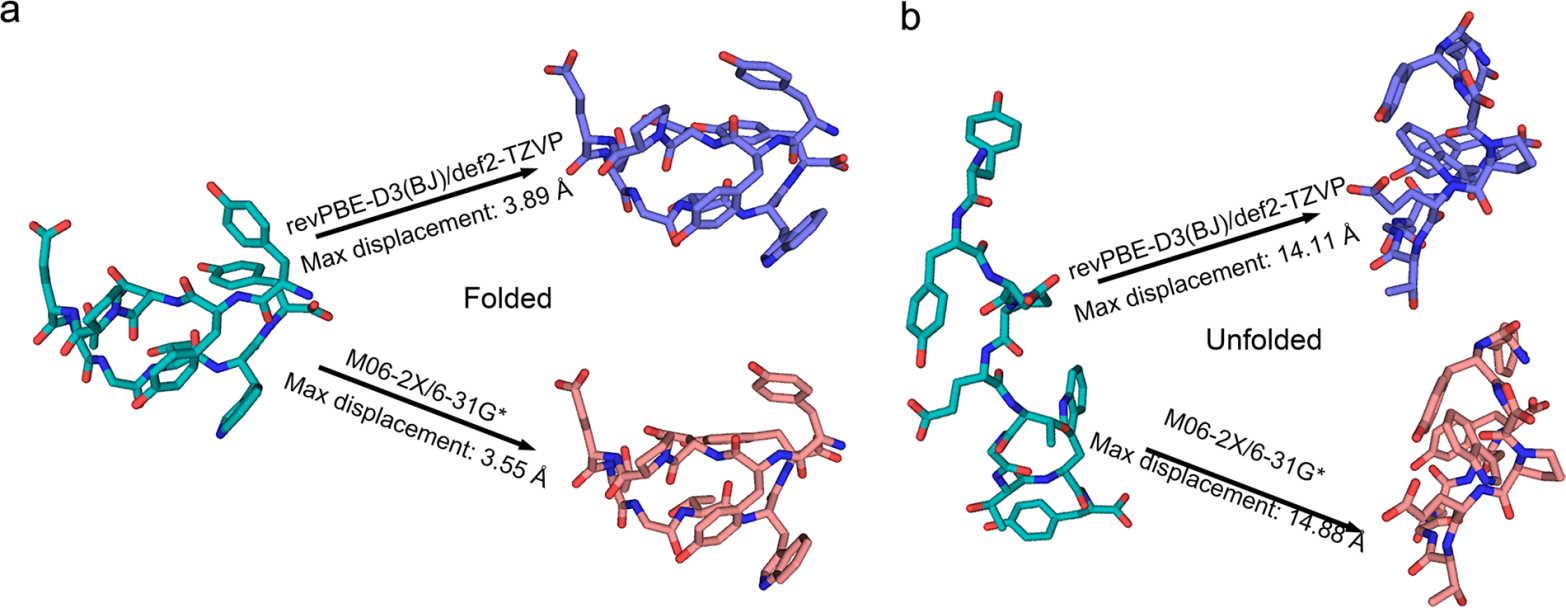
The result of geometry optimization from folded (**a**) or unfolded (**b**) *Chignolin* structure upon revPBE-D3(BJ)/def2-TZVP or M06-2X/6-31 G*. The maximum displacement of atoms on the output structure compared with the initial structure is shown on the right side.

Furthermore, we made geometry optimization at the revPBE-D3(BJ)/def2-TZVP level of theory and compared this with M06-2X/6-31 G* in terms of the final optimized structures. Starting from the folded or unfolded structures respectively, the similar optimized structures were obtained by revPBE-D3(BJ)/def2-TZVP or M06-2X/6-31 G* (Fig. 9a,b). The maximum displacement according to the initial structure were similar (folded-revPBE-D3(BJ)/def2-TZVP: 3.89 Å; folded-M06-2X/6-31 G*: 3.55 Å; unfolded-revPBE-D3(BJ)/def2-TZVP: 14.11 Å; unfolded-M06-2X/6-31 G*: 14.88 Å). It further underscores the precision of M06-2X as the benchmarking calculation approach.

**Fig. 10:**
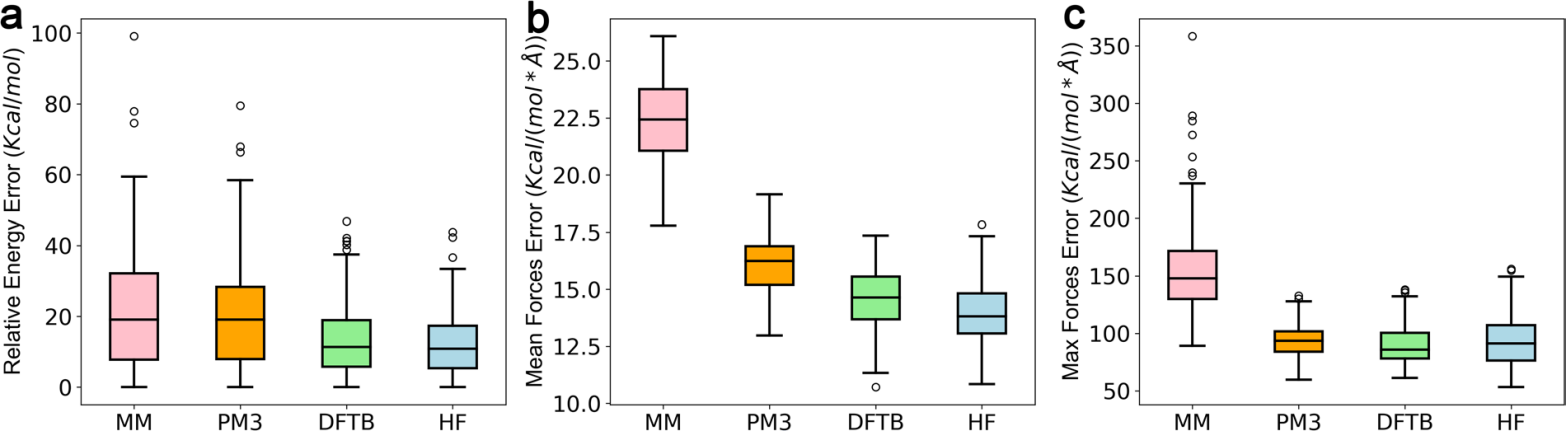
Evaluation of the calculation accuracy of molecular mechanics (MM), semi-empirical approach (PM3), Hartree-Fock (HF), Density Functional based Tight Binding (DFTB) and Density Functional Theory (DFT). (**a**) the mean absolute error of the potential energy; (**b**) the mean absolute error of atomic forces; (**c**) the maximum error of atomic forces. The values calculated by DFT that are used to construct the AIMD-Chig dataset are set as the ground truth values. The differences between the values calculated by MM, PM3 DFTB and HF and those calculated by DFT are shown in the boxplot and colored red, orange, green and blue, respectively. 200 samples are used for evaluation. In panel (**a**), the energy values subtracted the energy of a reference structure.

We also compared our DFT based approach with other lightweight approaches. Molecular mechanics (MM) is a widely common method for biomolecule conformational sampling. Semi-empirical (e.g., PM3 and DFTB) or Hartree-Fork (HF) are sometimes also employed for molecular dynamics simulations for biomolecules^1,19^. We compared the accuracy on 200 conformations sampled from the AIMD-Chig dataset using molecular mechanics (MM), semi-empirical approach comprising the NDDO approximation-based (PM3) and DFT approximation-based methods (DFTB), Hartree-Fock (HF), and Density Functional Theory (DFT) (Fig. 10, Table 3). We treat the energy and forces calculated by DFT as the ground truth values and evaluated the differences from those calculated by other approaches. For comparison on energy, we adopted the structure with the lowest energy as the reference and calculated the relative energies of other structures to it.

**Table 3:** Evaluation of calculation time and accuracy of different approaches.

Method	Energy error (kcal/mol)	Mean force error (kcal/mol/Å)	Max force error (kcal/mol/Å)	Time (s) (on 1 CPU thread)
MM	21.72 ± 17.17	22.28 ± 1.87	155.50 ± 39.24	0.013 ± 0.01
PM3	20.55 ± 15.26	16.07 ± 1.20	94.23 ± 14.24	4.86 ± 1.41
DFTB	13.76 ± 10.27	14.52 ± 1.33	89.28 ± 16.83	69.39 ± 35.63
HF	12.43 ± 8.97	13.97 ± 1.34	93.72 ± 21.20	30578.48 ± 4982.21
DFT	—	—	—	24873.84 ± 7862.77

The energy and forces calculated by DFT are set as the ground truth values.

As shown in Fig. 10a, MM had the most difference in energy (21.72 ± 17.17 kcal/mol) compared with the value calculated by DFT. The NDDO approximation-based semi-empirical approach, PM3, performed similar energy difference with MM (20.55 ± 15.26 kcal/mol) while DFT approximation-based semi-empirical approach DFTB (13.76 ± 10.27 kcal/mol) and HF performed much better (12.43 ± 8.97 kcal/mol). As for the mean absolute error of forces shown in Fig. 10b, MM still made a poor calculation (22.28 ± 1.87 kcal/mol/Å). As a comparison, PM3, DFTB and HF achieved differences of 16.07 ± 1.20 kcal/mol/Å, 14.52 ± 1.33 kcal/mol/Å and 13.97 ± 1.34 kcal/mol/Å, respectively. Both are closer to the ground truth. The maximum error of atomic forces was higher than the mean one. For MM, the value is 155.50 ± 39.24 kcal/mol/Å. PM3, DFTB and HF have their maximum error of 94.23 ± 14.24 kcal/mol/Å, 89.28 ± 16.83 kcal/mol/Å and 93.72 ± 21.20 kcal/mol/Å, respectively. It infers that PM3, DFTB and HF have similar max force error.

It is worth noticing that the energy and force errors are negatively related to the calculation time (Table 3). Although with an Intel Xeon Platinum 8272CL CPU core with a single thread, MM only took 0.01 seconds for each sample calculation. PM3 (4.86 ± 1.41 s) took hundreds of times longer than MM and the accuracy also increased. DFTB cost 69.39 ± 35.63 s per calculation and achieved similar accuracy with HF, showing its capability as a modern algorithm. Although both HF and DFT employed the same 6–31 G* basis and required a similar time scale with more than 10,000 seconds on one CPU thread, the gaps of energies and forces calculated by HF or DFT still existed. Therefore, given the high accuracy and tolerable cost, employing DFT level calculation to build the AIMD-Chig dataset is a reasonable choice.

## Data Availability

We employed ORCA 4.2.1 to run *ab initio* MD simulations and perform calculation accuracy comparisons between PM3 and HF approaches^40^ as well as DFTB + 22.2 for DFTB approach^51^. We used the Amber20 sander to run REMD simulations and perform calculation accuracy comparisons on MM. We also employed Amber20 pmemd.cuda for conventional MD simulations^54,55^. We used mdtraj 1.9.7 and MSMBuilder 3.8.0 for trajectory analysis and anchor selection^56,57^. We applied pytorch 1.13 and torch-geometric 2.0.4 for the training of VisNet. The time course and distribution analysis were drawn by seaborn 0.11.2. The free energy surfaces were generated via MATLAB R2019a.
